# YTHDF2 promotes anaplastic thyroid cancer progression by activating the DDIT4/AKT/mTOR signaling pathway

**DOI:** 10.1186/s13062-024-00566-y

**Published:** 2024-11-26

**Authors:** Bao Dai, Lei Xu, Shikuo Rong, Muye Song, Ziteng Lan, Weijian Chen, Lingyun Zhang, Yongchen Liu, Linhe Wang, Jinghua Li, Jian Chen, Zeyu Wu

**Affiliations:** 1 Department of Thyroid and Hernia Surgery, Guangdong Provincial People’s Hospital, Guangdong Academy of Medical Sciences, Southern Medical University, Guangzhou, Guangdong 510080 China; 2 Guangdong Cardiovascular Institute, Guangdong Provincial People’s Hospital, Guangdong Academy of Medical Sciences, Guangzhou, China; 3 Division of Thyroid surgery, Department of General Surgery, Shenzhen People’s Hospital (The First Affiliated Hospital, Southern University of Science and Technology; The Second Clinical Medical College, Jinan University ), Shenzhen, Guangdong China; 4 Department of Anesthesiology, Guangdong Provincial People’s Hospital Guangdong Academy of Medical Sciences, Southern Medical University, Guangzhou, Guangdong 510080 China; 5 Department of Laboratory, Guangdong Provincial People’s Hospital Guangdong Academy of Medical Sciences, Southern Medical University, 106 Zhongshan 2nd Road, Guangzhou, Guangdong 510080 China

**Keywords:** Anaplastic thyroid cancer, m6A, YTHDF2, AKT, mTOR

## Abstract

**Background:**

RNA methylation, an important reversible post-transcriptional modification in eukaryotes, has emerged as a prevalent epigenetic alteration. However, the role of the m6A reader YTH domain family 2 (YTHDF2) has not been reported in anaplastic thyroid cancer (ATC) and its biological mechanism is unclear.

**Methods:**

The relationship between YTHDF2 expression and ATC was determined using data sets and tissue samples. A range of analytical techniques were employed to investigate the regulatory mechanism of YTHDF2 in ATC, including bioinformatics analysis, m6A dot-blot analysis, methylated RNA immunoprecipitation sequencing (MeRIP-seq), RNA immunoprecipitation (RIP) assays, RNA sequencing, RNA stability assays and dual luciferase reporter gene assays. In vitro and in vivo assays were also conducted to determine the contribution of YTHDF2 to ATC development.

**Results:**

YTHDF2 expression was significantly increased in ATC. The comprehensive in vitro and in vivo experiments demonstrated that YTHDF2 knockdown significantly attenuated ATC proliferation, invasion, migration, and apoptosis promotion, whereas YTHDF2 overexpression yielded the opposite trend. Mechanistically, RNA-seq, MeRIP-seq and RIP-seq analysis, and molecular biology experiments demonstrated that YTHDF2 accelerated the degradation of DNA damage-inducible transcript 4 or regulated in DNA damage and development 1 (DDIT4, or REDD1) mRNA in an m6A-dependent manner, which in turn activated the AKT/mTOR signaling pathway and induced activation of epithelial-mesenchymal transition (EMT), thereby promoting ATC tumor progression.

**Conclusions:**

This study is the first to demonstrate that elevated YTHDF2 expression levels suppress DDIT4 expression in an m6A-dependent manner and activate the AKT/mTOR signaling pathway, thereby promoting ATC progression. YTHDF2 plays a pivotal role in ATC progression, and it may serve as a promising therapeutic target in the future.

**Supplementary Information:**

The online version contains supplementary material available at 10.1186/s13062-024-00566-y.

## Introduction

Thyroid cancer is the most prevalent malignant endocrine tumor, with an increasing incidence rate over recent years. It ranks as the ninth most common cancer worldwide and the third most common cancer in China [1, 2]. ATC is a rare but highly malignant form of thyroid cancer, accounting for 1–2% of all thyroid cancers and most thyroid cancer-related deaths [3, 4]. The American Joint Committee on Cancer tumor-lymph node-metastasis classification system generally classifies patients with ATC patients as stage IV [5]. High invasiveness and lethality are the distinguishing features of ATC, where the median overall survival from the date of diagnosis is 4 months, and the disease-specific mortality is nearly 100% [6]. More than 40% of patients with ATC present with distant metastases, which often result in death from local tumor expansion or asphyxiation due to distant metastasis [7]. Currently, there is no effective treatment to extend the overall survival time [3, 8]. However, understanding the ATC molecular landscape, particularly the characterization of such drivers of carcinogenesis in malignancies, has identified potential opportunities for targeted therapies [9].

m6A is the most common internal modification of eukaryotic mRNA, and is dynamically reversible and widely involved in mRNA splicing, nuclear output, stability and translation [10, 11]. m6A methylases, demethylases, and recognition proteins regulate this modification [12, 13]. Alterations in the expression of these proteins can affect tumor transcripts and oncoprotein expression, cancer cell proliferation, survival, tumorigenesis, progression, metastasis and sensitivity to anticancer therapies [14–17]. YTHDF2, the earliest binding recognition protein, regulates the stability of m6A-modified mRNAs by recognizing and binding to m6A-modified sites to degrade target genes [18, 19], This process promotes the progression of different tumors, as evidenced by studies in which YTHDF2 was upregulated in tumor cells and promoted tumor growth and metastasis [20–23]. Furthermore, the absence of YTHDF2 in tumor-associated macrophages (TAMs) impedes tumor growth by reprogramming TAMs to an antitumor phenotype and enhancing their capacity to cross-present antigens. These findings suggested that inhibiting YTHDF2 may enhance the efficacy of cancer immunotherapy [24]. Nevertheless, the role and molecular mechanism of YTHDF2 in ATC remain incompletely understood.

In the process of metastasis, cells undergo epithelial-mesenchymal transition (EMT), which involves the loss of epithelial cell surface markers and the acquisition of characteristics associated with mesenchymal cells. These cells are involved in the distant metastasis of tumors, as evidenced by studies [25, 26]. Recent studies have indicated that m6A modification regulates EMT and its transcription factors [27, 28]. Furthermore, intervention with m6A modification demonstrated the potential for cancer therapy [29]. Previous studies have demonstrated that the mTOR signaling pathway is crucial in tumor progression. For example, in triple-negative breast cancer, EGFR influences the EMT marker MMP2 by activating the PI3K/AKT/mTOR pathway, while MMP9 expression facilitates tumor metastasis [30]. GPRC5A inhibits the ubiquitination-dependent degradation of LAMTOR1, which leads to the recruitment of mTORC1 to lysosomes and activation of the mTORC1/p70S6K signaling pathway. This process promotes docetaxel resistance and liver metastasis [31]. Furthermore, miR-25-3p/PHLPP2/PI3K-AKT signaling pathway activation in hypoxic glioma cells enhances M2 macrophage polarization, thereby advancing glioma progression [32]. Additionally, AKT/mTOR pathway has been closely associated with EMT; for example, in liver cancer and non-small cell lung cancer, mTOR signaling pathway activation induces EMT and subsequently accelerates tumor progression [33, 34].

In the present study, the biological functions of YTHDF2 and its potential molecular mechanisms in the pathogenesis of human ATC were delineated using in vitro and in vivo experiments. Our results indicate that YTHDF2 activates the AKT/mTOR signaling pathway by degrading DDIT4, thereby promoting ATC proliferation, migration, and invasion.

## Materials and methods

### Specimens from patients

From June 2014 to December 2023, human thyroid cancer and its adjacent tissues were collected from the Guangdong General Hospital for formalin fixation and paraffin embedding. The thyroid cancer were diagnosed by histopathology. These clinical materials were used for research purposes with the patients’ prior consent of the patients and institutional research ethics committee approval.

### Cell lines and cell culture

The ATC cell lines (BHT101, KHM-5 M and KMH2) and follicular thyroid cancer cell line (FTC133) were purchased from Wuhan Pricella Biotechnology Co., Ltd. The thyroid cancer cell lines (CAL-62) and the normal thyroid cell line (Nthy-ori 3 − 1) were purchased from the Beijing Bina Biological BNCC cell bank, and all were confirmed by short tandem repeat analysis. The KHM-5 M and Nthy-ori 3 − 1 cells were cultured in RPMI1640(Gibco, USA) medium containing 10% fetal bovine serum (FBS, Procell, Wuhan, China). The FTC133 cells are cultured in a medium consisting of 10% fetal bovine serum in a 1:1 mixture of RPMI1640(Gibco, USA) medium and Dulbecco’s modified Eagle’s medium (DMEM, Gibco) medium. The CAL-62, BHT101 and FTC133 cell lines were cultured in 10% DMEM (Gibco, USA) medium at 37 °C and 5% CO_2_ in the cell incubator.

### Immunohistochemistry (IHC) and hematoxylin and eosin (H&E) staining

The paraffin-embedded tissues were sectioned at 5 μm thickness. The sections were deparaffinized using xylene, rehydrated with a gradient alcohol series, then incubated for 30 min with 3% hydrogen peroxide at room temperature. Antigen retrieval was achieved through microwave heating in sodium citrate buffer. Finally, the sections were blocked with 5% bovine serum albumin (BSA), then incubated overnight with anti-YTHDF2 (Proteintech, China)at 4 °C. The sections were then treated for 30 min with peroxidase-labeled streptavidin at room temperature after the addition of the polymer enhancer. The antibody reaction was observed with a fresh substrate solution containing 3,3’-diaminobenzidine tetrahydrochloride (DAB). The paraffin sections were deparaffinized and hematoxylin stained for three minutes. The sections should then be rinsed with running water and stained with eosin for 30 s–1 min. The stained sections were then examined under a microscope.

### Immunofluorescence staining

The appropriate cells were added to confocal dishes at 37 °C overnight, then fixed with 4% paraformaldehyde for 20 min and permeated with 0.3% Triton X-100 for 10 min. The cells were then blocked with 5% BSA for 30 min and incubated overnight with anti-YTHDF2 (Proteintech, China) and anti-m6A (Proteintech, China) antibodies at 4 °C. Subsequently, the cells were incubated with a secondary antibody for 1 h at room temperature. A cytoskeleton fluorescence assay was conducted using a cytoskeleton red fluorescence probe (KeyGEN Biotech, China) on cells that had been blocked at room temperature for 20 min. The nuclei were then stained with DAPI for a period of 5 min. Subsequently, fluorescent images were captured using a confocal fluorescence microscope.

### Cell counting Kit-8 (CCK-8) assay

Cell proliferation was measured using CCK-8(Beyotime, China). 2000/well cells (6 double wells) were seeded on 96-well plates at 37 °C and adhered to the wall overnight. At the designated time point, the culture medium in six replicate wells was replaced with 100 µL of fresh medium containing 10% CCK-8 reagent.

### 5-ethynyl-20-deoxyuridine (EdU) assay

Cell proliferation was quantified using an EdU assay kit (Ribobio, Guangzhou, China). The cells (10 × 10^5^ cells per well) were seeded into 6-well plates and incubated overnight. The following day, the cells were incubated with 50 µM EdU buffer at 37 °C for 2 h, fixed with 4% paraformaldehyde for 0.5 h, and permeabilized with 0.5% Triton X-100 for 10 min in flow tubes. Each tube was stained with 1× Apollo and incubated at room temperature for 10 min in the dark. The supernatant was then discarded, and the cells were resuspended in 500 µL of phosphate-buffered saline(PBS). Flow cytometry was then employed to detect the cells.

### Trans-well invasion assay

The matrix gel was diluted in accordance with the instructions and applied to an 8-µm pore size trans-well filter insert in a 24-well plate for the invasion assay. The cells (5 × 10^4^) were placed in the upper chamber in serum-free medium, while 10% FBS medium was added to the lower chamber. Following a 36-hour incubation period at 37 °C, the cells situated beneath the membrane were fixed and stained with crystal violet (Beyotime, China). The penetrated cells were counted in five random fields under the microscope.

### Wound healing assay

Cells (5 × 10^5^ cells per well) were seeded in 6-well plates at 37 °C overnight. The cells were then replaced with serum-free medium and a 200 µL pipette tip was used to create a scratch. The migration rate of cells was quantified by microscopy at 0 h and 36 h post-scratch. All tests were conducted in triplicate.

### RNA extraction and quantitative real-time PCR (qPCR)

Total RNA was extracted from cells using ESScience RNA Rapid Extraction Kit (YiShan Biotech, Shanghai, China) according to the manufacturer’s instructions, and quantified with a NanoDrop 2000 unit. The complementary DNA was generated from 1 µg RNA and real-time qPCR was performed to determine determine the levels of the target RNA. The data were analyzed according to the ΔCt method. The primers utilized in this study are presented in Supplementary Table S2.

### Western blotting (WB)

Cells and animal tissue were lysed in radioimmunoprecipitation assay (RIPA) lysis buffer (New Cell and Molecular Biotech, Suzhou, China) supplemented with protease and phosphatase inhibitors to extract the protein. The protein lysates were separated by sodium dodecyl sulfate-polyacrylamide gel electrophoresis (SDS-PAGE) and transferred onto 0.45 μm polyvinylidene difluoride membranes (Millipore, USA). The membranes were then incubated in Tris-buffered saline with Tween-20 (TBST) containing 5% bovine serum albumin (BSA) for 1 h at room temperature. Subsequently, the membranes were incubated with primary antibodies overnight at 4 °C. The primary antibodies were then detected with secondary antibodies conjugated with horseradish peroxidase. The immunoblots were imaged using an imaging system (Bio-Rad, USA) and the ECL western blotting kit (Millipore, USA). The antibodies utilized were detailed in Supplementary Table S3.

### m6A dot-blot analysis

Total RNA was extracted using the RN001 RNA Quick Purification kit(ESScience, Shanghai, China), and quantified with a NanoDrop 2000. Then 2 µL of the RNA sample was applied to the positively-charged nylon transfer membrane (BersinBio, Guangzhou, China) and subjected to UV crosslinking twice to the nylon transfer membrane. Subsequently, the membrane was blocked with 5% BSA, followed by an overnight incubation with the anti-m6A antibody at 4 °C. After incubating with the corresponding second antibody for 1 h, the membrane was developed and the blots were visualized using an ECL western blotting kit (Millipore).

### RNA immunoprecipitation (RIP) assays

The RIP assays were performed according to the manufacturer’s instructions (Beyotime, Shanghai, China). The cell lysate was divided into three samples: anti-YTHDF2, anti-IgG, and input. Immunoprecipitation was performed overnight at 4 °C using 5 µg specific antibody or IgG as the negative control. The levels of the target RNA were quantified using qPCR.

### Transient transfection with pDDIT4

The overexpression plasmid pDDIT4 and the control pNull (empty vector) (Miaoling Biology, Wuhan, China) were transfected using the Lipofectamine™ 3000 transfection reagent (Invitrogen, USA) according to the manufacturer’s protocol.

### Lentivirus vector infection

Lentiviral constructs expressing YTHDF2 and green fluorescent protein (GFP), and the negative control lentivirus, were purchased from Hanbio (Shanghai, China). The sequences of the lentiviruses that overexpress YTHDF2 (YTHDF2), YTHDF2-shRNA (shYTHDF2-1, shYTHDF2-2), the empty vector (Vec), and the scrambled shRNA (shNC) are listed in Supplementary Table S1. We have detected the expression level of YTHDF2 protein remains relatively low in BHT101 cells and relatively higher in CAL-62 cells among all ATC cell lines.CAL-62 cells were used to establish YTHDF2 knockdown cell line. BHT101 cells were selected to establish stable YTHDF2 overexpression cell lines. Transfection was conducted in accordance with the manufacturer’s instructions. Briefly, 5 × 10^4 cells were plated into a 6-well plate and transfected with the indicated lentivirus. Infected cells were selected using 2 µg/ml puromycin (MCE, USA) for ≥ 1 week, and the transfection efficiency was determined by qPCR and western blotting.

### Dual-luciferase reporter assay

Wild-type and mutant segments were synthesized using m6A motifs in DDIT4-5ʹ untranslated region (5’UTR) (A-T). The indicated cells were transfected with DDIT4 wild-type or mutant dual-luciferase reporter plasmid. At 48 h post-transfection, the luciferase activities were detected using a Dual-Lumi™ II Luciferase Reporter Gene Assay Kit (Beyotime, Shanghai, China).

### RNA decay assays

After 0-, 1-, 2-, and 3-h treatment with 5 µg/mL actinomycin D, total RNA was extracted from the CAL-62 or BHT101 cells using the RNA Quick Purification Kit (ESScience, Shanghai, China). The expression levels were quantified by qPCR analysis.

### Methylated RIP sequencing (MeRIP-seq) and MeRIP-qPCR

Total RNA was isolated and purified using TRIzol (Invitrogen, USA) following the manufacturer’s instructions. Poly (A) RNA was purified from 50 µg total RNA using Dynabeads Oligo (dT)25-61005 (Thermo Fisher Scientific, USA) through two rounds of purification. Subsequently, the poly(A) RNA was fragmented for 7 min using the Magnesium RNA Fragmentation Module (cat. e6150, NEB, USA) at 86℃. The cleaved RNA fragments were incubated with m6A-specific antibody for immunoprecipitation. The input RNA samples and immunoprecipitated RNA samples were prepared for subsequent sequencing procedures or qPCR analyses.

### Animal experiments

Provincial People’s Hospital Ethics Review Committee approved all animal experiments (acceptance No. S2023-357-02.20160006). For the purpose of establishing tumor xenograft models, 5 × 10^6 CAL-62 shYTHDF2-1 or BHT101 OEYTHDF2 cells, along with empty vector cells, suspended in 200 µl of PBS were separately implanted into the right flank of 4-week-old BALB/c mic which were randomly divided into 4 groups (4 mice per group). The volumes of the tumors (V = width^2 × length × 0.52) were recorded at 4-day intervals using a caliper. The mice were euthanized at 28 days post-implantation, and the tumors were photographed and weighed. Transfected (1 × 10^6) cells suspended in 100 µL of PBS were injected into the tail vein of BALB/c nude mice (4 weeks old), which were randomly divided into 4 groups (5 mice per group). One month after the injection, the average radiation efficiency (p/s/cm^2/sr) was quantified by an in vivo imaging system (IVIS), after which the mice were sacrificed. The GFP-positive tissues were excised and underwent IHC and H&E staining.

### Statistical analysis

The data are reported as the mean ± standard deviation (SD). Differences between groups were assessed using, Student’s t-test, a non-parametric test (Mann-Whitney test), or a one-way analysis of variance (ANOVA) with Bonferroni’s correction was employed. All analyses were conducted using GraphPad Prism 9.0, A two-tailed value of *P < 0.05, **P < 0.01, and ***P < 0.001 was considered statistically significant.

## Results

### YTHDF2 and m6A expression in ATC

**Fig. 1:**
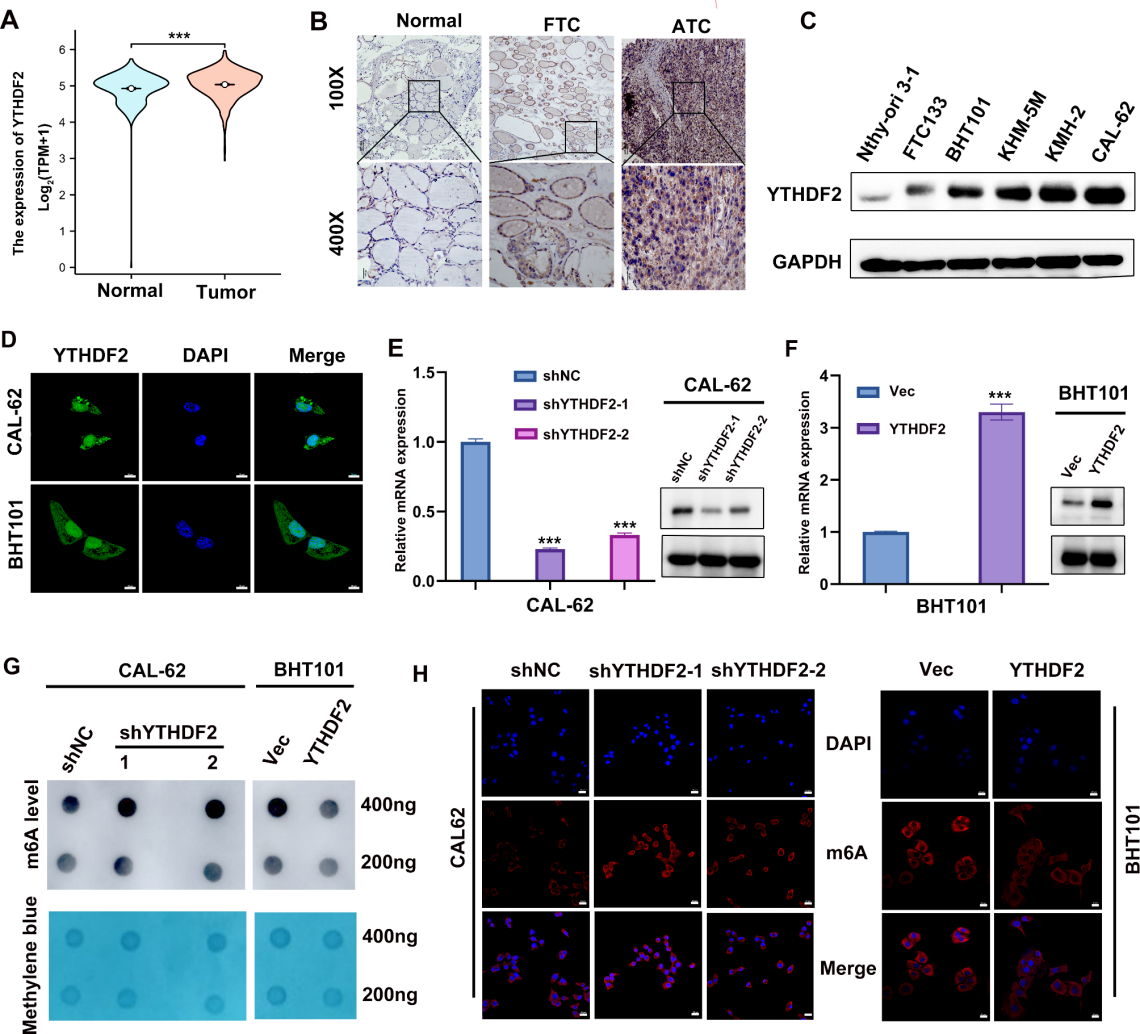
YTHDF2 and m6A expression levels in ATC. **A** The YTHDF2 expression pattern were analyzed in 512 thyroid tumors and 338 normal controls (UCSC XENA database). The Mann-Whitney test was used for statistical analysis. **B** Representative IHC staining images of YTHDF2 expression in ATC, FTC and normal thyroid tissues. **C** YTHDF2 protein expression levels in normal thyroid, FTC and ATC cell lines. **D** Immunofluorescence detection of the subcellular localization of YTHDF2, scale bar = 10 μm. **E**, **F** YTHDF2 was knocked down and overexpressed in CAL-62 and BHT101 cells, respectively, and the efficiency was verified by qPCR and WB. GAPDH was the internal reference. **G** m6A RNA dot-blot assay. m6A level changes were detected in CAL-62 and BHT101 cell lines at different total RNA concentrations (200 ng, 400 ng). Methylene blue staining was used as loading control. **H** Immunofluorescence detection of m6A levels in CAL-62 and BHT101 cell lines. Scale bar = 20 μm. Error bars represent the SD obtained from at least three independent experiments. *P < 0.05, **P < 0.01, ***P < 0.001

To investigate the expression pattern of YTHDF2 in thyroid cancer, UCSC XENA (https://xenabrowser.net/datapages/) was utilized. This revealed that YTHDF2 was up-regulated between thyroid carcinoma tissues and normal tissues (Fig. 1A). Furthermore, the level of YTHDF2 protein was found to be increased in ATC paraffin-embedded specimens compared to FTC and normal specimens using an IHC assay (Fig. 1B). A comparable pattern was observed in the thyroid cancer subtype cell lines, including thyroid cancer cell lines (CAL-62, KMH-2, KHM-5 M, BHT101, FTC133), as well as a human normal thyroid cell line (Nthy-ori 3 − 1) (Fig. 1C). The immunofluorescence assay results indicated that YTHDF2 was distributed in the cytoplasm andnucleus (Fig. 1D). To identify the biological function of YTHDF2 in ATC, the endogenous expression of YTHDF2 was effectively knocked down in the CAL-62 cell line, and a stable YTHDF2-overexpressing transfection was established in the BHT101 cell line (Fig. 1E, F). Previous studies have demonstrated that YTHDF2 upregulates m6A levels by inducing targeted mRNA decay [35, 36]. The m6A level alterations were clarified using an RNAm6A dot blot assay, which revealed upregulated total m6A levels in the YTHDF2 knock-down cell lines when RNA concentrations varied (200 ng and 400 ng) (Fig. 1G). The cell immunofluorescence of m6A supported this result (Fig. 1H).

### YTHDF2 promoted ATC cells proliferation, invasion and migration in vitro

**Fig. 2:**
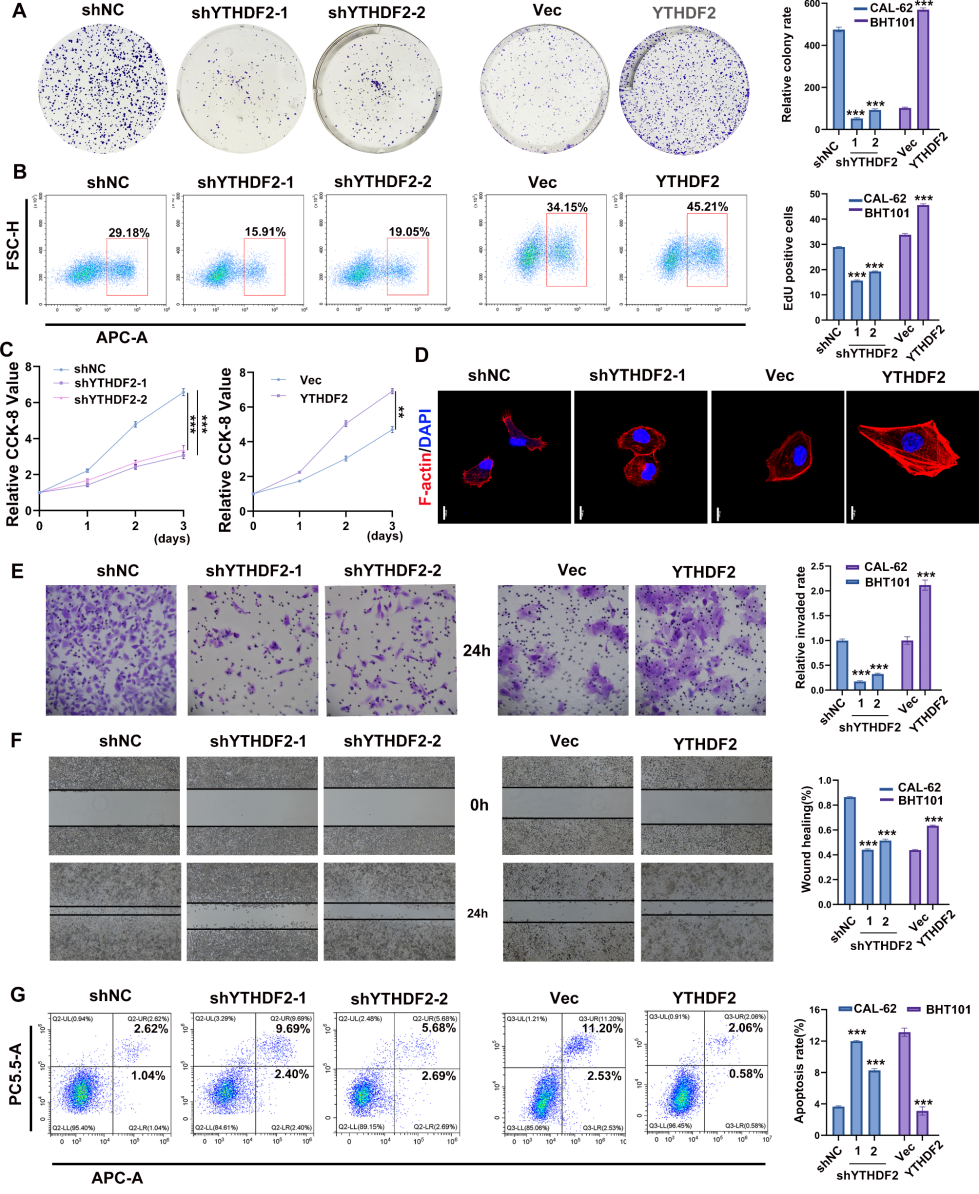
YTHDF2 promoted ATC malignant phenotype progression. **A-C** Effect of YTHDF2 on ATC proliferation detected by clonal colony formation assay, EdU assay, and CCK-8 assay. **D** F-actin fluorescent staining measurement of the effect of YTHDF2 overexpression and knockdown on cell cytoskeleton. Representative images of the F-actin fluorescent staining are shown. Scale bar = 10 μm. **E**, **F** Trans-well assay and wound healing assay to verify the effect of YTHDF2 knockdown and overexpression on cell migration ability. **G** Flow cytometry assay to confirm the apoptosis analysis induced by YTHDF2 knock-down and overexpression. Error bars represent the SD from at least three independent experiments. *P < 0.05, **P < 0.01, ***P < 0.001

To investigate the role of YTHDF2 in ATC tumorigenesis, we conducted a colony formation assay. The results revealed that YTHDF2 knockdown significantly reduced the CAL-62 colony formation rates, whereas YTHDF2 overexpression increased the BHT101 colony formation rates (Fig. 2A). The EdU assay results indicated that YTHDF2 knockdown suppressed the ability of DNA replication in CAL-62 cells, as the YTHDF2 knockdown group had a decreased percentage of EdU-positive cells compared with the control group. Conversely, overexpressing YTHDF2 had the opposite effect (Fig. 2B). The results of the CCK-8 assay were also consistent with those of the EdU assay (Fig. 2C). Actin–microtubule crosstalk plays a pivotal role in regulating cell shape and polarity during cell migration and division [37, 38]. Therefore, we analyzed the cytoskeleton using F-actin fluorescent staining. Compared to the control cells, the YTHDF2 knock-down cells exhibited a cobblestone-like shape and shrinkable F-actin fibers, which impeded cell migration, cellular morphology elongation, and F-actin fibers stretching. Conversely, the cells overexpressing YTHDF2exhibited elongated cellular morphology and stretched F-actin fibers, which are well-suited for cellular migration (Fig. 2D and Supplementary Fig. S1). The Transwell assay revealed that the cell invasion rate of the YTHDF2-deficient group was significantly reduced compared to that of the control group after 36 h incubation in serum-free medium. Contrastingly, the opposite effect was observed in the cells overexpressing YTHDF2 (Fig. 2E). Furthermore, the wound healing assay demonstrated that knocking down YTHDF2 impeded the cell migration rate (Fig. 2F). A further flow cytometry assay of two cell lines indicated that YTHDF2 knock-down had an inhibitory effect on cell apoptosis. However, YTHDF2 overexpression appeared to attenuate the apoptosis rate (Fig. 2G). Collectively, these results ndicated that YTHDF2 promoted the proliferation, migration, and invasion of ATC cells.

### YTHDF2 induced AKT/mTOR activation and EMT by suppressing DDIT4 expression

**Fig. 3:**
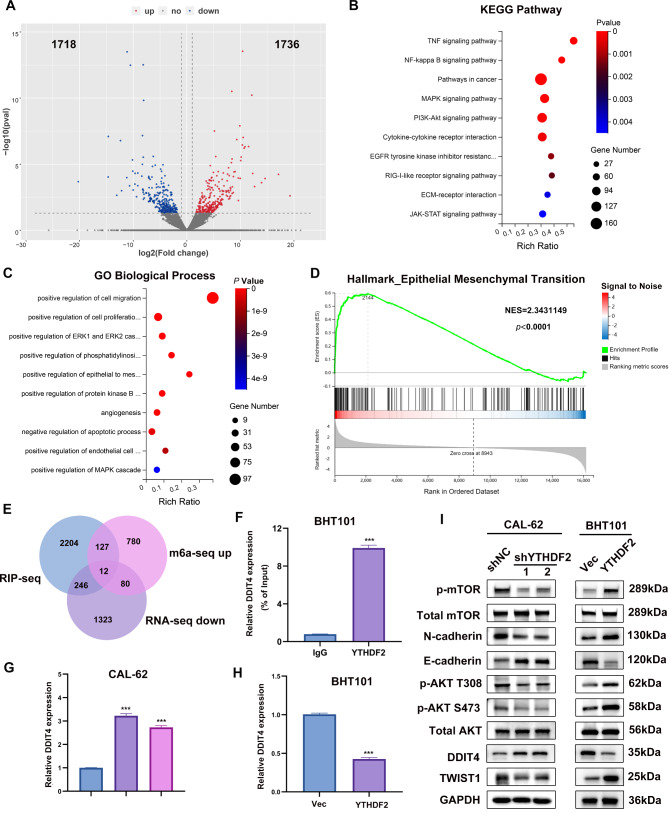
YTHDF2 facilitated the ATC progression by activating the AKT-mTOR pathway and EMT by suppressing DDIT4 expression. **A** Fold change in differential expression of genes in different subgroups of MeRIP (shNC, shYTHDF2) was represented by log2(foldchange) as the horizontal coordinate. Red color represents up-regulated significant differential expression (1736 genes) and blue color represents down-regulated significant differential expression (1718 genes). **B**, **C** KEGG analysis and GO-BP enrichment analysis were performed on the differential genes (|log_2_FC| > 0.5, *P* < 0.05). **D** GSEA revealing that the YTHDF2-related differential genes are significantly enriched in the EMT pathway. **E** Venn diagram depicting the intersecting genes from the MeRIP-seq, RIP-seq and mRNA-seq correlated genes. Twelve common genes were screened out. **F** RIP-qPCR confirmation of the DDIT4 mRNA enrichment by YTHDF2 in BHT cell line. **G**-**I** qPCR and WB verification of DDIT4 expression levels. I Validation of the effect of YTHDF2 on AKT/mTOR and EMT pathway by WB. Error bars represent the SD from at least three independent experiments; *P < 0.05, **P < 0.01, and ***P < 0.001

To investigate the potential and direct m6A-modified targets of YTHDF2, we conducted a MeRIP-seq in YTHDF2 stably knocked down CAL-62 cell line (CAL-62 shYTHDF2) and its control (CAL-62 shNC). The volcano plot indicated that the m6A levels of 1736 genes were upregulated and 1718 genes were downregulated after knocking down YTHDF2 (Fig. 3A). The biological function and molecular mechanism of YTHDF2 in ATC was studied using Kyoto Encyclopedia of Genes and Genomes (KEGG) analysis and Gene Ontology biological process (GO-BP) enrichment analysis of the differential genes (|log_2_ fold change [FC]| > 0.5, *P* < 0.05). The TNF, NF-κB and PI3K/AKT pathways were significantly enriched and closely related to biological functions such as proliferation, invasion and metastasis (Fig. 3B and C). Gene set enrichment analysis (GSEA) revealed that YTHDF2-related highly expressed genes were significantly enriched in the EMT pathway (Fig. 3D). To identify YTHDF2 target genes, RNA-seq, MeRIP-seq, and RIP-seq were combined, focusing on genes with high m6A levels and significant changes in mRNA expression. This approach yielded 12 genes (Fig. 3E).

Given that the most typical role of YTHDF2 is to accelerate the degradation of mRNAs by recognizing m6A modifications [18, 35], we hypothesized that YTHDF2 might activate the EMT-associated pathway by degrading negative regulatory genes. Through a literature review, we found that DDIT4 has been identified as a negative regulator of mTORC1 and mTORC2, acting as a central signal transduction hub involved in cellular metabolism, energy homeostasis, and cell growth networks [39–42]. To assess the impact of YTHDF2 knockdown on DDIT4 mRNA binding, YTHDF2 RIP-qPCR was conducted. The results showed that YTHDF2 enrichment of DDIT4 mRNA was diminished in the cells overexpressing YTHDF2 (Fig. 3F). YTHDF2 exhibited a significant negative regulatory effect on DDIT4 mRNA levels in CAL-62 and BHT101 cells, as evidenced by qPCR and Western blot analysis (Fig. 3G-I). Knocking down YTHDF2 downregulated that EMT-related proteins and inhibited AKT phosphorylation, while overexpressing YTHDF2 yielded the opposite trend (Fig. 3I).

### YTHDF2 mediated DDIT4 mRNA degradation in an m6A-dependent manner

**Fig. 4:**
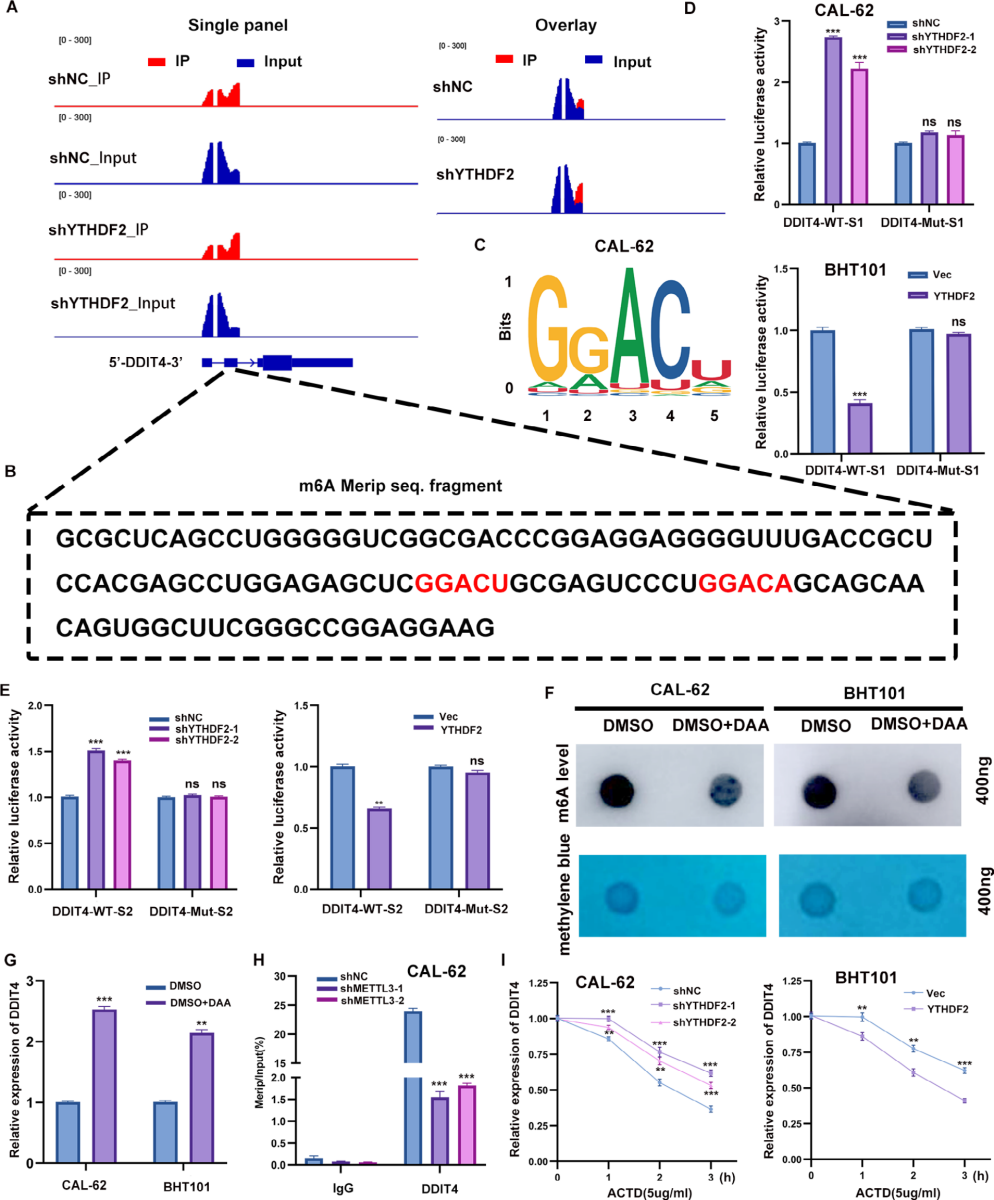
YTHDF2 mediated the DDIT4 mRNA degradation in an m6A-dependent manner. **A** IGV plots showed the m6A and YTHDF2 binding site and the change of RNA-seq peaks at DDIT4 mRNAs in CAL-62. **B** The sequence represents the fragments captured in MeRIP-seq, which was co-localized with predicted sites. **C** The m6A peaks motifs are RRACH (R = G/A, H = A/C/U). **D** and E Relative luciferase activity of DDIT4 5’UTR with wild-type or mutated m6A sites after YTHDF2 knockdown and overexpression in CAL-62 and BHT101 cells. Renilla luciferase activity was measured and normalized to firefly luciferase activity. **F** The total RNA m6A level after DAA treatment was detected by m6A RNA dot blot assay and compared with DMSO treatment, with methylene blue staining as a loading control. **G** qPCR was used for the detection of DDIT4 mRNA levels after DAA treatment. **H** MeRIP-RT-qPCR detection of m6A level alterations of DDIT4 after METTL3 knockdown in CAL-62 cells. **I** RT-qPCR analysis of the decay rate of DDIT4 mRNA after actinomycin D (5 µg/mL) treatment in CAL-62 and BHT101 cells with YTHDF2knockdown or overexpression. Error bars represent the SD from at least three independent experiments. *P < 0.05, **P < 0.01, ***P < 0.001

To further validate the existence of m6A modification on DDIT4 mRNA in ATC, Integrative genomics viewer (IGV) plots demonstrated the m6A modification sites, the YTHDF2 binding sites and the change in MeRIP-seq peak of DDIT4 mRNA upon YTHDF2 knockdown in CAL-62 cell line. The results demonstrated the presence of peaks mapping to DDIT4 mRNA in m6A antibody-precipitated RNA, and reads of peaks mapping to DDIT4 mRNA were also increased in YTHDF2-knockdown cells (Fig. 4A). Two significantly upregulated m6A sites in 5’UTR of DDIT4 at single site resolution were identified in the YTHDF2 knock-down CAL-62 cell line from MeRIP-seq data analysis (Fig. 4B). The motifs of the m6A peaks were consistent with the consensus sequence [RRACH (R = G/A, H = A/C/U)] (Fig. 4C). Furthermore, a dual luciferase activity assay demonstrated that the knockdown of YTHDF2 significantly elevated luciferase activity in the wild-type group (GGACU/GGACA, m6A sites included in 5’UTR), but not in the mutated group (mutated m6A sites from A to T), while overexpressing YTHDF2 had the opposite effect (Fig. 4D, E).

To ascertain whether this regulation was m6A-dependent, we employed the global methylation inhibitor, 3-deazaadenosine (DAA), to treat the CAL-62 and BHT101 cell lines. Our findings revealed that the total m6A levels, as determined by RNA m6A dot blot assay, exhibited a notable reduction compared to the dimethyl sulfoxide (DMSO) control group at 400 ng RNA concentration (Fig. 4F). Consequently, the mRNA expression of DDIT4 was also upregulated with the same concentration of DAA treatment (Fig. 4G). Furthermore, MeRIP-qPCR demonstrated a considerable elevation in DDIT4 mRNA levels in the m6A antibody-precipitated RNA relative to that of IgG. Additionally, DDIT4 mRNA precipitated by the m6A antibody decreased markedly in Mettl3-knockdown CAL-62 cells (Fig. 4H). Subsequently, the effects of YTHDF2 knockdown and overexpression on the stability of DDIT4 mRNA was studied. The stability of DDIT4 mRNA was increased in CAL-62 cells following YTHDF2 knockdown and decreased in BHT101 cells overexpressing YTHDF2 (Fig. 4I).

Collectively, YTHDF2 facilitated DDIT4 mRNA degradation by recognizing m6A-modified sites, and this regulation was m6A-dependent.

### YTHDF2 accelerated tumor growth and metastasis in vivo

**Fig. 5:**
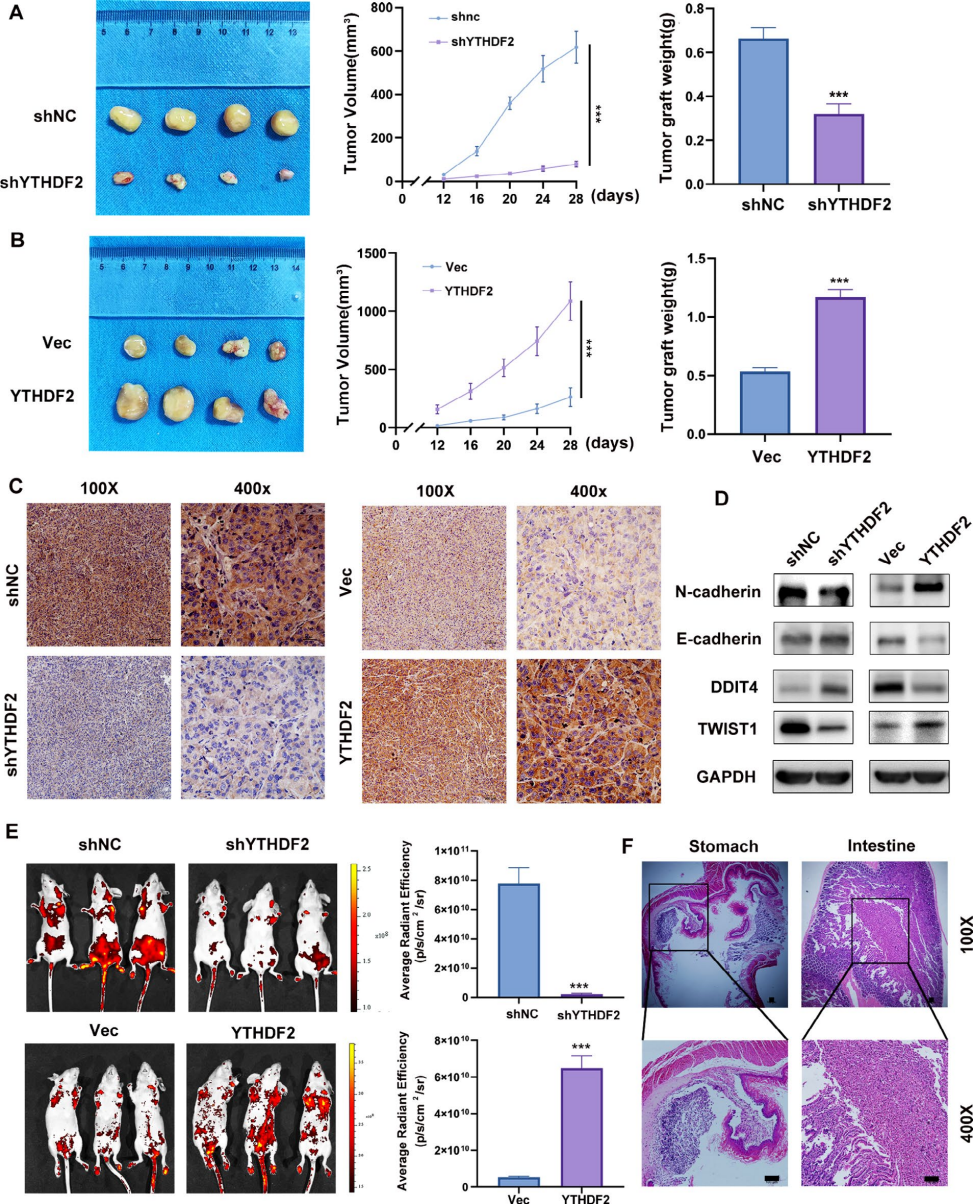
YTHDF2 promoted tumor growth and metastasis in vivo. **A**, **B** The tumor growth curve of xenografts was plotted by measuring the tumor size (width^2 × length × 0.52) with caliper. The tumor size at the endpoint in the above group was analyzed by Student’s t-test. The subcutaneous tumor models were observed after 28 days, the anatomized subcutaneous tumor xenografts were weighed and analyzed with Student’s t-test. **C** Representative IHC staining micrographs of YTHDF2 in tumor xenografts were conducted. **D**. Proteins were extracted from animal subcutaneous tumors for WB detection of EMT-associated proteins. **E**. The BALB/c nude mice injected with cells via tail vein were imaged at week 4 to evaluate the entire metastasis by the IVIS. **F**. H&E staining of multiple metastatic organs was performed to identify the metastatic loci. Error bars represent the SD obtained from at least three independent experiments.*P < 0.05, **P < 0.01, ***P < 0.001

To investigate the effect of YTHDF2 on the malignant phenotype in vivo, we also constructed stable YTHDF2 knockdown and overexpressing cells, expressing YTHDF2 and GFP constructed by lentivirus. The tumor size was observed and measured every 4 days until it reached an appropriate size. The results demonstrated that knocking down YTHDF2 resulted in a dramatic retardation of tumor growth and weight (Fig. 5A). Contrastingly, xenografts overexpressing YTHDF2 exhibited accelerated tumor progression (Fig. 5B). IHC staining of subcutaneous tumor tissues revealed that YTHDF2 expression levels were significantly lower in the shYTHDF2 group and significantly higher in the YTHDF2 overexpression group (Fig. 5C). Protein levels were verified by extracting subcutaneous tumor proteins, where the expression levels of the EMT-related proteins N-cadherin, TWIST1 were decreased and E-cadherin was increased in the shYTHDF2 group, while the opposite trend was shown in the YTHDF2 overexpression group (Fig. 5D). The extent of metastasis was assessed using a tail vein injection metastatic model. The results indicated that YTHDF2 knockdown significantly inhibited the metastasis of CAL-62 and BHT101 cells at week 4. This was further corroborated by the reduced radiance value measured by the IVIS after 28 days (Fig. 5E). The metastatic organs were then anatomized and imaged using the in vivo imaging system, which facilitated the precise localization and identification of the metastases. The above metastatic tissues were prepared as slides and stained with H&E to identify the metastatic sites (Fig. 5F). In conclusion, the results demonstrated that YTHDF2 significantly accelerated tumor growth and metastasis in vivo.

### DDIT4 overexpression reversed the influence of YTHDF2 on the malignant phenotype of tumors and AKT/mTOR activation

**Fig. 6:**
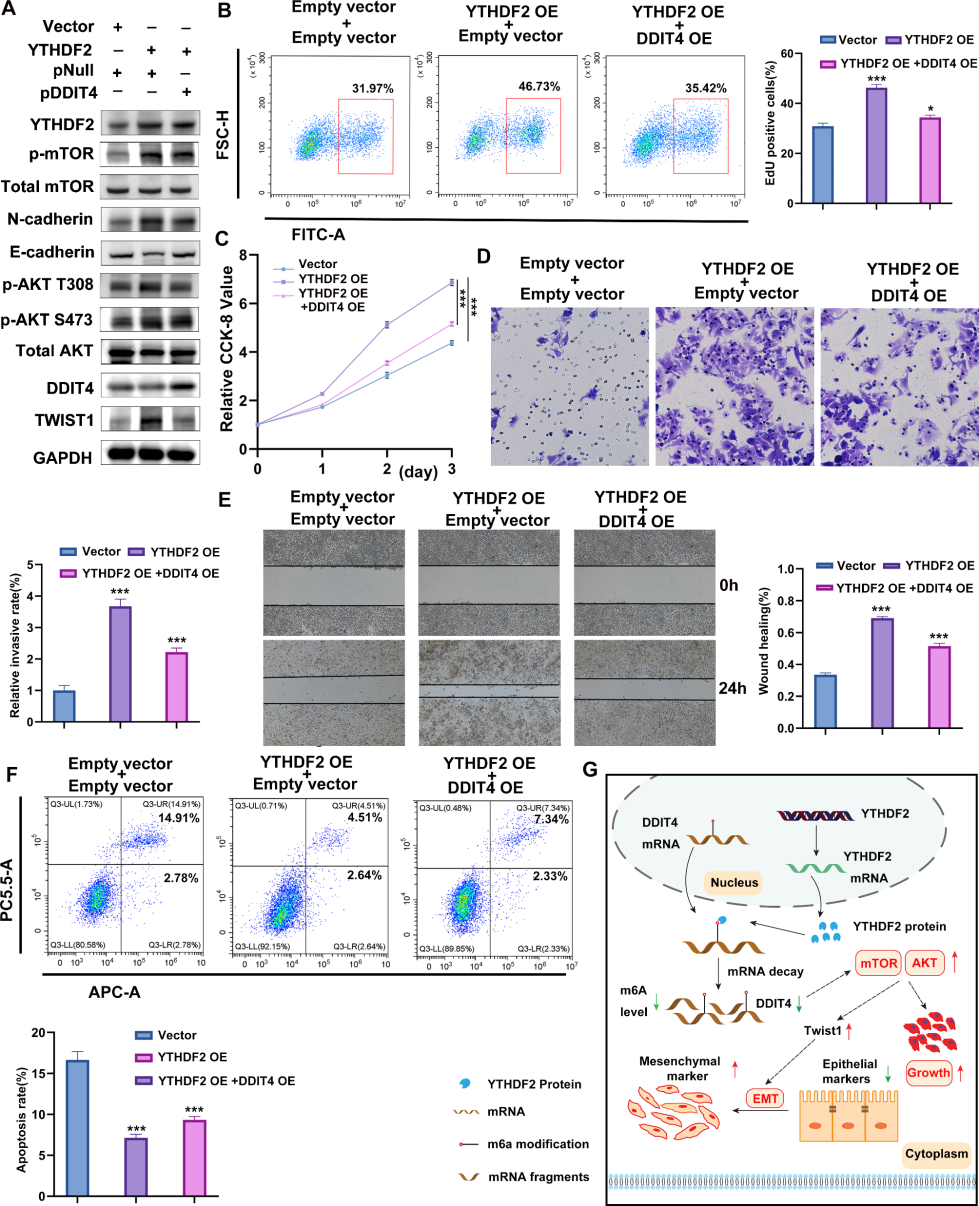
The tumor suppressor role of DDIT4 in ATC. **A** To verify the transfection efficiency of DDIT4 and detect the expression levels of AKT/mTOR and EMT-related proteins by WB. GAPDH was the internal reference. **B**, **C** EdU and CCK-8 assay evaluation of the proliferative capacity of cells after DDIT4 overexpression. **D**, **E** Trans-well and wound healing assay evaluation of cell migration upon DDIT4 overexpression. **F** Flow cytometry assay evaluation of the apoptosis induced by DDIT4 overexpression. **G** Schematic diagram representing the results of this study. Error bars represent the SD from at least three independent experiments. *P < 0.05, **P < 0.01, ***P < 0.001

The role of DDIT4 in YTHDF2 promotion of ATC progression and AKT/mTOR activation was validated by overexpressing DDIT4 in OEYTHDF2 cells. Western blotting demonstrated that DDIT4 overexpression suppressed AKT/mTOR activation and EMT-associated proteins induced by YTHDF2 overexpression (Fig. 6A). The CCK-8 and EdU assays indicated that DDIT4 overexpression significantly attenuated the proliferation of OEYTHDF2 BHT101 cells(Fig. 6B, C). Meanwhile, wound-healing assay and trans-well migration analysis showed the DDIT4 overexpression reduced the increased migration of BHT101 cells caused by YTHDF2 overexpression (Fig. 6D, E). Flow cytometry revealed that DDIT4 attenuated the effect of YTHDF overexpression on apoptosis (Fig. 6F). These findings indicated that DDIT4 overexpression reversed the influence of YTHDF2 on the malignant phenotype of tumors and AKT-mTOR activation. Therefore, we determined YTHDF2 exerted its degradation function by targeting the tumor suppressor DDIT4, consequently activating the DDIT4/AKT/mTOR signaling pathway and inducing EMT in ATC. All these results were summarized in a schematic diagram (Fig. 6G).

## Discussion

RNA methylation, an important reversible post-transcriptional modification in eukaryotes, has emerged as a broad regulatory mechanism with implications in almost all significant biological processes, including the initiation and progression of cancer [10, 19, 43, 44]. The potential therapeutic value of YTHDF2 is being explored in ATC treatment. The most recent study reported that deletingYTHDF2 in a knockout mouse model after ionizing radiation altered the differentiation of myeloid-derived suppressor cells, inhibit their transport to the tumor, and weaken their suppressive function, thereby enhancing anti-tumor immunity and the effectiveness of local tumor irradiation [45]. Oxaliplatin upregulates YTHDF2 expression by activating the cGAS-STING signaling pathway. YTHDF2 stabilizes the CX3CL1 transcript in an manner dependent on m6A, modulating the interaction between CD8 T cells and thereby promoting treatment-induced anti-tumor immune responses in the liver [46]. YTHDF2 can also promote immune evasion and angiogenesis in liver cancer by recognizing the m6A modification site in the 5’UTR of ETS variant transcription factor 5 mRNA and recruiting the eukaryotic translation initiation factor 3 subunit B to facilitate its translation [47]. These studies indicate that inhibiting YTHDF2 can enhance anti-tumor immunity and the effects of radiotherapy, making YTHDF2 a promising new target for clinical radiotherapy.

Furthermore, chemical intervention has identified and optimized a small molecule inhibitor of YTHDF2, DC-Y13-27, through chemical intervention. This compound can directly bind to the YTHDF2 protein and inhibit its binding to m6A-RNA in vitro. In mouse models, DC-Y13-27 has shown effects similar to Ythdf2 knockout, effectively enhancing the efficacy of IR and the anti-tumor effects of IR combined with PD-L1 antibody treatment [48]. YTHDF2 also promotes the progression of diffuse large B-cell lymphoma by regulating alkaline ceramidase 2-mediated ceramide metabolism in an m6A-dependent manner [49]. These studies not only reveal the multiple roles of YTHDF2 in tumor development but also provide a scientific basis for the development of new targeted therapeutic strategies. In this study, YTHDF2 was found to promote ATC progression by recognizing the binding 5’UTR m6A modification site and accelerating the degradation of the target gene DDIT4.

Previous studies reported that the AKT/mTOR signaling pathway was closely related to tumor proliferation and metastasis [30, 31, 50], and participated in the EMT process of tumors [33, 34, 51, 52]. ATC is characterized by the rapid proliferation of local lesions, high rate of invasion and metastasis, and poor prognosis [6]. ATC displays a more active epithelial-mesenchymal transition than papillary thyroid carcinoma, which enhances its invasion and metastasis outside the thyroid [53]. Furthermore, we reviewed the literature and determined DDIT4 has been identified as a potent mTORC1 pathway inhibitor, regulating various cellular processes, including protein synthesis, autophagy, and cell growth [54, 55]. Previous studies have found that up-regulation of DDIT4 in human myeloma cells inhibits AKT and mTOR signaling and exerts anti-tumor progression effects [56]. Moreover, DDIT4 was suggested to facilitate bladder urothelial carcinoma (BUC) progression and antagonized REDD1, thereby sensitizing BUC cells to paclitaxel [57]. In the present study, overexpression of DDIT4 significantly reduced the phosphorylation of AKT and mTOR. Further experiments have confirmed that DDIT4 is the direct target of YTHDF2. To confirm the dependence of this regulation on m6A, demethylation of ATC cells by DAA demonstrated a significant decrease in m6A levels and an increase in DDIT4 RNA expression. Moreover, the knockdown of METTL3 levels in CAL-62 cell lines yielded consistent results. This further substantiates the conclusion that the aforementioned regulation is m6A-dependent. Consequently, we hypothesized that YTHDF2 induces the degradation of DDIT4 by binding to m6A modification sites, which was confirmed by dual luciferase experiments on GGACU/GGACA in the 5’UTR, respectively.

Previous studies suggested that DDIT4 promoted protein phosphatase 2 A-dependent dephosphorylation of AKT on Thr308 but not Ser473 [39], and that TSC2 phosphorylation required AKT phosphorylation on Thr308, but not Ser473 [58]. We observed that the AKT-mTOR pathway was activated following YTHDF2-mediated degradation of the DDIT4 target gene. Concurrently, the expression level of EMT-related proteins was significantly elevated. Western blot experiments revealed that following the decrease in the expression of DDIT4, the phosphorylation levels of the main phosphorylation sites of AKT, Thr308 and Ser473, were increased. Of these, the change in the phosphorylation level of Thr308 was more correlated to the change in DDIT4. It is possible that DDIT4 more significantly affects the AKT Thr308 phosphorylation site. and this hypothesis was consistent with the findings of the aforementioned study.

Previous studies have found that the TWIST1 transcription factor can promote tumor metastasis and induce EMT [59, 60]. TWIST1 upregulation of SOX2 was associated with the promotion of cancer stem cell-like properties in prostate cancer cells and also with the progression of triple-negative breast cancer [61, 62]. Our results indicated that the TWIST1 protein expression level was consistent with that of EMT-related proteins and YTHDF2, but opposite to that of DDIT4. This results indicated that YTHDF2 may additionally influence the EMT progression by regulating TWIST1, where discovering the underlying mechanisms requires further research.

RNA methylation is widely involved in numerous biological processes, yet little has been reported in ATC. In the present study, we determined for the first time that YTHDF2 is important in ATC However, given the small number of ATC cases and the extremely poor prognosis, valid clinical data to verify the clinical significance of our study. Future research should explore the potential application and clinical translation value of YTHDF2 in ATC treatment, such as using liposomes with an affinity for ATC to target YTHDF2, and investigating its effects when used in combination with radiotherapy or chemotherapy in ATC cells, animal models, and ATC organoids.

## Conclusions

Our study demonstrates for the first time that elevated YTHDF2 expression levels inhibit DDIT4 expression levels in an m6A-dependent manner and activate the AKT/mTOR signaling pathway to promote ATC proliferation, metastasis, and inhibit apoptosis. These findings indicate that YTHDF2 plays a pivotal role in ATC progression and may serve as a promising therapeutic target.

## Electronic supplementary material

Below is the link to the electronic supplementary material.

Supplementary Material 1

## Data Availability

No datasets were generated or analysed during the current study.

## Abbreviations

ATC: Anaplastic Thyroid Cancer
YTHDF2: YTH domain family 2
RIP: RNA immunoprecipitation
m6A: N6-methyladenosine
EMT: Epithelial-mesenchymal transition
AJCC: The American Joint Committee on Cancer
TNM: Tumor-lymph node-metastasis
TAM: Tumor-associated macrophage
DDIT4, or REDD1: DNA damage and Development 1
FBS: Fetal bovine serum
IHC: Immunohistochemistry
H&E: Hematoxylin and Eosin
BSA: Bovine serum albumin
DAB: 3,3′-diaminobenzidine tetrahydrochloride
CCK-8: The Cell Counting Kit-8
EdU: 5-ethynyl-20-deoxyuridine
DAA: 3-deazaadenosine
