# Supplementary material for: YTHDF2 promotes anaplastic thyroid cancer progression by activating the DDIT4/AKT/mTOR signaling pathway
Source: Biol Direct. 2024 Nov 26;19:122. doi: 10.1186/s13062-024-00566-y (PMC11600618; doi:10.1186/s13062-024-00566-y)
Supplement: Supplementary file 1 — Supplementary Material 1 [file 13062_2024_566_MOESM1_ESM.docx]

**Supplementary table S1. qPCR primer sequence**

| **Primer name^a^** | **Primer sequence** |
| --- | --- |
| YTHDF2-F | CCTTAGGTGGAGCCATGATTG |
| YTHDF2-R | TCTGTGCTACCCAACTTCAGT |
| DDIT4-F | AGGACGCACTTGTCTTAGCAG |
| DDIT4-R | GCAAGGACGAGGGCGAAGAG |
| GAPDH-F | AGAAGGCTGGGGCTCATTTG |
| GAPDH-R | AGGGGCCATCCACAGTCTTC |
| METTL3-F | CTCTGCGGGAGAGGCTGCAG |
| METTL3-R | GAGAAGAAGTTGCTACACCAC |
| ^b^Me-DDIT4-R | GGCAGGACGCACTTGTCTTA |
| ^b^Me-DDIT4-R | CTCTGTCTTGGAGGGCACTG |

a: F, forward primer; R, reverse primer.

b: Primers used in MeRIP qPCR.

**Supplementary table S2. Antibodies and Information**

| **Antibodies** | **Information** |
| --- | --- |
| YTHDF2 | 1:1000(WB)/1:200(IF), Abcam, USA |
| GAPDH | 1:1000(WB), Proteintech, China |
| DDIT4 | 1:1000(WB), Proteintech, China |
| Total mTOR | 1:1000(WB), Proteintech, China |
| p-mTOR | 1:1000(WB), Cell Signaling Technology, USA |
| Total AKT | 1:1000(WB), Proteintech, China |
| p-AKT308 | 1:1000(WB), Cell Signaling Technology, USA |
| p-AKT473 | 1:1000(WB), Cell Signaling Technology, USA |
| E-cadherin | 1:1000(WB), Proteintech, China |
| N-cadherin | 1:1000(WB), Proteintech, China |
| Twist1 | 1:1000(WB), Proteintech, China |
| m6A Monoclonal antibody | 1:1000(WB, Dot Blot)/ (1:200,IF), Proteintech, China |
| Multi-rAb HRP-Goat Anti-Rabbit Recombinant Secondary Antibody (H+L) | 1:5000(WB), Proteintech, China |
| Multi-rAb HRP-Goat Anti-Mouse Recombinant Secondary Antibody (H+L) | 1:5000(WB), Proteintech, China |
| Multi-rAb CoraLite® Plus 594-Goat Anti-Rabbit Recombinant Secondary Antibody (H+L) | 1:500(IF), Proteintech, China |
| Multi-rAb CoraLite® Plus 594-Goat Anti-Mouse Recombinant Secondary Antibody (H+L) | 1:500(IF), Proteintech, China |

**Supplementary table S3. Sequences of shRNA. and pRNA**

| Name | **Sequences** |
| --- | --- |
| shNC^a^ | TTCTCCGAACGTGTCACGTAA |
| shYTHDF2-1^a^ | GAGCCCTAGGTAGCACTCCATTTCT |
| shYTHDF2-2^a^ | GGCTGGTTCTGGATCTACTCCTTCA |
| YTHDF2^b^ | Atgtcggccagcagcctcttggagcagagaccaaaaggtcaaggaaacaaagtacaaaatggatctgtacatcaaaaggatggattaaacgatgatgattttgaaccttacttgagtccacaggcaaggcccaataatgcatatactgccatgtcagattcctacttacccagttactacagtccctccattggcttctcctattctttgggtgaagctgcttggtctacggggggtgacacagccatgccctacttaacttcttatggacagctgagcaacggagagccccacttcctaccagatgcaatgtttgggcaaccaggagccctaggtagcactccatttcttggtcagcatggttttaatttctttcccagtgggattgacttctcagcatggggaaataacagttctcagggacagtctactcagagctctggatatagtagcaattatgcttatgcacctagctccttaggtggagccatgattgatggacagtcagcttttgccaatgagaccctcaataaggctcctggcatgaatactatagaccaagggatggcagcactgaagttgggtagcacagaagttgcaagcaatgttccaaaagttgtaggttctgctgttggtagcgggtccattactagtaacatcgtggcttccaatagtttgcctccagccaccattgctcctccaaaaccagcatcttgggctgatattgctagcaagcctgcaaaacagcaacctaaactgaagaccaagaatggcattgcagggtcaagtcttccgccacccccgataaagcataacatggatattggaacttgggataacaagggtcccgttgcaaaagccccctcacaggctttggttcagaatataggtcagccaacccaggggtctcctcagcctgtaggtcagcaggctaacaatagcccaccagtggctcaggcatcagtagggcaacagacacagccattgcctccacctccaccacagcctgcccagctttcagtccagcaacaggcagctcagccaacccgctgggtagcacctcggaaccgtggcagtgggttcggtcataatggggtggatggtaatggagtaggacagtctcaggctggttctggatctactccttcagaaccccacccagtgttggagaagcttcggtccattaataactataaccccaaagattttgactggaatctgaaacatggccgggttttcatcattaagagctactctgaggacgatattcaccgttccattaagtataatatttggtgcagcacagagcatggtaacaagagactggatgctgcttatcgttccatgaacgggaaaggccccgtttacttacttttcagtgtcaacggcagtggacacttctgtggcgtggcagaaatgaaatctgctgtggactacaacacatgtgcaggtgtgtggtcccaggacaaatggaagggtcgttttgatgtcaggtggatttttgtgaaggacgttcccaatagccaactgcgacacattcgcctagagaacaacgagaataaaccagtgaccaactctagggacactcaggaagtgcctctggaaaaggctaagcaggtgttgaaaattatagccagctacaagcacaccacttccatttttgatgacttctcacactatgagaaacgccaagaggaagaagaaagtgttaaaaaggaacgtcaaggtcgtgggaaataa |
| shMETTL3-1^a^ | CCUGCAAGUAUGUUCACUA |
| shMETTL3-2^a^ | GCUACCUGGACGUCAGUAU |
| pDDIT4^c^ | gacggatcgggagatctcccgatcccctatggtgcactctcagtacaatctgctctgatgccgcatagttaagccagtatctgctccctgcttgtgtgttggaggtcgctgagtagtgcgcgagcaaaatttaagctacaacaaggcaaggcttgaccgacaattgcatgaagaatctgcttagggttaggcgttttgcgctgcttcgcgatgtacgggccagatatacgcgttgacattgattattgactagttattaatagtaatcaattacggggtcattagttcatagcccatatatggagttccgcgttacataacttacggtaaatggcccgcctggctgaccgcccaacgacccccgcccattgacgtcaataatgacgtatgttcccatagtaacgccaatagggactttccattgacgtcaatgggtggagtatttacggtaaactgcccacttggcagtacatcaagtgtatcatatgccaagtacgccccctattgacgtcaatgacggtaaatggcccgcctggcattatgcccagtacatgaccttatgggactttcctacttggcagtacatctacgtattagtcatcgctattaccatggtgatgcggttttggcagtacatcaatgggcgtggatagcggtttgactcacggggatttccaagtctccaccccattgacgtcaatgggagtttgttttggcaccaaaatcaacgggactttccaaaatgtcgtaacaactccgccccattgacgcaaatgggcggtaggcgtgtacggtgggaggtctatataagcagagctctctggctaactagagaacccactgcttactggcttatcgaaattaatacgactcactatagggagacccaagctggctagcgtttaaacttaagccaccatggactacaaagaccatgacggtgattataaagatcatgacatcgactacaaggatgacgatgacaagcttggtaccgagctcggatccatgcctagcctttgggaccgcttctcgtcgtcgtccacctcctcttcgccctcgtccttgccccgaactcccaccccagatcggccgccgcgctcagcctgggggtcggcgacccgggaggaggggtttgaccgctccacgagcctggagagctcggactgcgagtccctggacagcagcaacagtggcttcgggccggaggaagacacggcttacctggatggggtgtcgttgcccgacttcgagctgctcagtgaccctgaggatgaacacttgtgtgccaacctgatgcagctgctgcaggagagcctggcccaggcgcggctgggctctcgacgccctgcgcgcctgctgatgcctagccagttggtaagccaggtgggcaaagaactactgcgcctggcctacagcgagccgtgcggcctgcggggggcgctgctggacgtctgcgtggagcagggcaagagctgccacagcgtgggccagctggcactcgaccccagcctggtgcccaccttccagctgaccctcgtgctgcgcctggactcacgactctggcccaagatccaggggctgtttagctccgccaactctcccttcctccctggcttcagccagtccctgacgctgagcactggcttccgagtcatcaagaagaagctgtacagctcggaacagctgctcattgaggagtgttgagaattctgcagatatccagcacagtggcggccgctcgagtctagagggcccgtttaaacccgctgatcagcctcgactgtgccttctagttgccagccatctgttgtttgcccctcccccgtgccttccttgaccctggaaggtgccactcccactgtcctttcctaataaaatgaggaaattgcatcgcattgtctgagtaggtgtcattctattctggggggtggggtggggcaggacagcaagggggaggattgggaagacaatagcaggcatgctggggatgcggtgggctctatggcttctgaggcggaaagaaccagctggggctctagggggtatccccacgcgccctgtagcggcgcattaagcgcggcgggtgtggtggttacgcgcagcgtgaccgctacacttgccagcgccctagcgcccgctcctttcgctttcttcccttcctttctcgccacgttcgccggctttccccgtcaagctctaaatcgggggctccctttagggttccgatttagtgctttacggcacctcgaccccaaaaaacttgattagggtgatggttcacgtagtgggccatcgccctgatagacggtttttcgccctttgacgttggagtccacgttctttaatagtggactcttgttccaaactggaacaacactcaaccctatctcggtctattcttttgatttataagggattttgccgatttcggcctattggttaaaaaatgagctgatttaacaaaaatttaacgcgaattaattctgtggaatgtgtgtcagttagggtgtggaaagtccccaggctccccagcaggcagaagtatgcaaagcatgcatctcaattagtcagcaaccaggtgtggaaagtccccaggctccccagcaggcagaagtatgcaaagcatgcatctcaattagtcagcaaccatagtcccgcccctaactccgcccatcccgcccctaactccgcccagttccgcccattctccgccccatggctgactaattttttttatttatgcagaggccgaggccgcctctgcctctgagctattccagaagtagtgaggaggcttttttggaggcctaggcttttgcaaaaagctcccgggagcttgtatatccattttcggatctgatcaagagacaggatgaggatcgtttcgcatgattgaacaagatggattgcacgcaggttctccggccgcttgggtggagaggctattcggctatgactgggcacaacagacaatcggctgctctgatgccgccgtgttccggctgtcagcgcaggggcgcccggttctttttgtcaagaccgacctgtccggtgccctgaatgaactgcaggacgaggcagcgcggctatcgtggctggccacgacgggcgttccttgcgcagctgtgctcgacgttgtcactgaagcgggaagggactggctgctattgggcgaagtgccggggcaggatctcctgtcatctcaccttgctcctgccgagaaagtatccatcatggctgatgcaatgcggcggctgcatacgcttgatccggctacctgcccattcgaccaccaagcgaaacatcgcatcgagcgagcacgtactcggatggaagccggtcttgtcgatcaggatgatctggacgaagagcatcaggggctcgcgccagccgaactgttcgccaggctcaaggcgcgcatgcccgacggcgaggatctcgtcgtgacccatggcgatgcctgcttgccgaatatcatggtggaaaatggccgcttttctggattcatcgactgtggccggctgggtgtggcggaccgctatcaggacatagcgttggctacccgtgatattgctgaagagcttggcggcgaatgggctgaccgcttcctcgtgctttacggtatcgccgctcccgattcgcagcgcatcgccttctatcgccttcttgacgagttcttctgagcgggactctggggttcgaaatgaccgaccaagcgacgcccaacctgccatcacgagatttcgattccaccgccgccttctatgaaaggttgggcttcggaatcgttttccgggacgccggctggatgatcctccagcgcggggatctcatgctggagttcttcgcccaccccaacttgtttattgcagcttataatggttacaaataaagcaatagcatcacaaatttcacaaataaagcatttttttcactgcattctagttgtggtttgtccaaactcatcaatgtatcttatcatgtctgtataccgtcgacctctagctagagcttggcgtaatcatggtcatagctgtttcctgtgtgaaattgttatccgctcacaattccacacaacatacgagccggaagcataaagtgtaaagcctggggtgcctaatgagtgagctaactcacattaattgcgttgcgctcactgcccgctttccagtcgggaaacctgtcgtgccagctgcattaatgaatcggccaacgcgcggggagaggcggtttgcgtattgggcgctcttccgcttcctcgctcactgactcgctgcgctcggtcgttcggctgcggcgagcggtatcagctcactcaaaggcggtaatacggttatccacagaatcaggggataacgcaggaaagaacatgtgagcaaaaggccagcaaaaggccaggaaccgtaaaaaggccgcgttgctggcgtttttccataggctccgcccccctgacgagcatcacaaaaatcgacgctcaagtcagaggtggcgaaacccgacaggactataaagataccaggcgtttccccctggaagctccctcgtgcgctctcctgttccgaccctgccgcttaccggatacctgtccgcctttctcccttcgggaagcgtggcgctttctcatagctcacgctgtaggtatctcagttcggtgtaggtcgttcgctccaagctgggctgtgtgcacgaaccccccgttcagcccgaccgctgcgccttatccggtaactatcgtcttgagtccaacccggtaagacacgacttatcgccactggcagcagccactggtaacaggattagcagagcgaggtatgtaggcggtgctacagagttcttgaagtggtggcctaactacggctacactagaagaacagtatttggtatctgcgctctgctgaagccagttaccttcggaaaaagagttggtagctcttgatccggcaaacaaaccaccgctggtagcggtttttttgtttgcaagcagcagattacgcgcagaaaaaaaggatctcaagaagatcctttgatcttttctacggggtctgacgctcagtggaacgaaaactcacgttaagggattttggtcatgagattatcaaaaaggatcttcacctagatccttttaaattaaaaatgaagttttaaatcaatctaaagtatatatgagtaaacttggtctgacagttaccaatgcttaatcagtgaggcacctatctcagcgatctgtctatttcgttcatccatagttgcctgactccccgtcgtgtagataactacgatacgggagggcttaccatctggccccagtgctgcaatgataccgcgagacccacgctcaccggctccagatttatcagcaataaaccagccagccggaagggccgagcgcagaagtggtcctgcaactttatccgcctccatccagtctattaattgttgccgggaagctagagtaagtagttcgccagttaatagtttgcgcaacgttgttgccattgctacaggcatcgtggtgtcacgctcgtcgtttggtatggcttcattcagctccggttcccaacgatcaaggcgagttacatgatcccccatgttgtgcaaaaaagcggttagctccttcggtcctccgatcgttgtcagaagtaagttggccgcagtgttatcactcatggttatggcagcactgcataattctcttactgtcatgccatccgtaagatgcttttctgtgactggtgagtactcaaccaagtcattctgagaatagtgtatgcggcgaccgagttgctcttgcccggcgtcaatacgggataataccgcgccacatagcagaactttaaaagtgctcatcattggaaaacgttcttcggggcgaaaactctcaaggatcttaccgctgttgagatccagttcgatgtaacccactcgtgcacccaactgatcttcagcatcttttactttcaccagcgtttctgggtgagcaaaaacaggaaggcaaaatgccgcaaaaaagggaataagggcgacacggaaatgttgaatactcatactcttcctttttcaatattattgaagcatttatcagggttattgtctcatgagcggatacatatttgaatgtatttagaaaaataaacaaataggggttccgcgcacatttccccgaaaagtgccacctgacgtc |

a: lentivirus vector: pHBLV-U6-MCS-CMV-ZsGreen-PGK-PURO

b: pHBLV-CMV-MCS-3FLAG-EF1-ZsGreen-T2A-PURO

c: pECMV-3×FLAG-Neo/G418

**Fig. S1**

**
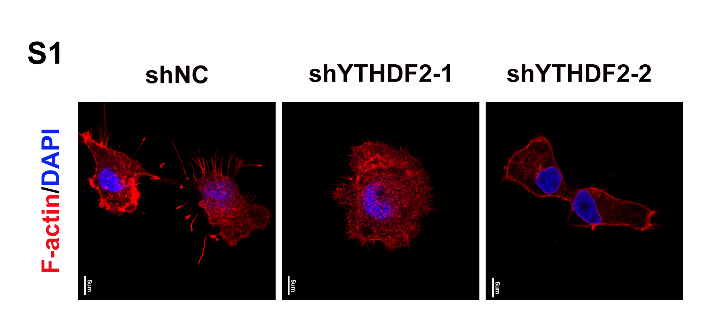
**

**Fig.S1** Effect of YTHDF2 knockdown on the CAL-62 cytoskeleton.

**S1** The effect of knockdown of YTHDF2 on CAL-62 cells cytoskeleton was measured by F-actin fluorescent staining. Representative images of the F-actin fluorescent staining are shown, scale bar = 5 μm.
